# Mixtures of Biological Control Agents and Organic Additives Improve Physiological Behavior in Cape Gooseberry Plants under Vascular Wilt Disease

**DOI:** 10.3390/plants10102059

**Published:** 2021-09-29

**Authors:** José Luis Chaves-Gómez, Cristian Camilo Chávez-Arias, Alba Marina Cotes Prado, Sandra Gómez-Caro, Hermann Restrepo-Díaz

**Affiliations:** 1 Departamento de Agronomía, Facultad de Ciencias Agrarias, Universidad Nacional de Colombia, Sede Bogotá, Carrera 30 No. 45-03, Bogotá 111321, Colombia; jlchavesgo@unal.edu.co (J.L.C.-G.); ccchaveza@unal.edu.co (C.C.C.-A.); sgomezc@unal.edu.co (S.G.-C.); 2 Corporación Colombiana de Investigación Agropecuaria-AGROSAVIA, Centro de Investigación Tibaitatá, Km 14 vía Bogotá a Mosquera, Mosquera 250047, Colombia; amcotes@agrosavia.com

**Keywords:** Andean fruit species, *Bacillus velezensis*, burnt rice husk, chitosan, *Fusarium oxysporum*, *Trichoderma virens*

## Abstract

This study aimed to assess the soil application of mixtures of biological control agents (BCAs) (*Trichoderma virens* and *Bacillus velezensis*) and organic additives (chitosan and burnt rice husk) on the physiological and biochemical behavior of cape gooseberry plants exposed to *Fusarium oxysporum* f. sp. *physali* (Foph) inoculum. The treatments with inoculated and non-inoculated plants were: (i) *T. virens* + *B. velezensis* (Mix), (ii) *T. virens* + *B. velezensis* + burnt rice husk (MixRh), (iii) *T. virens* + *B. velezensis* + chitosan (MixChi), and (iv) controls (plants without any mixtures). Plants inoculated and treated with Mix or MixChi reduced the area under the disease progress curve (AUDPC) (57.1) and disease severity index (DSI) (2.97) compared to inoculated plants without any treatment (69.3 for AUDPC and 3.2 for DSI). Additionally, these groups of plants (Mix or MixChi) obtained greater leaf water potential (~−0.5 Mpa) and a lower MDA production (~12.5 µmol g^−2^ FW) than plants with Foph and without mixtures (−0.61 Mpa and 18.2 µmol g^−2^ FW, respectively). The results suggest that MixChi treatments may be a promising alternative for vascular wilt management in cape gooseberry crops affected by this disease.

## 1. Introduction

Cape gooseberry (*Physalis peruviana* L.) is a plant species belonging to the Solanaceae family and its center of origin is the Andean region of South America [1]. This fruit has acquired economic importance due to its high content of vitamins A, C, and B, essential minerals such as iron (Fe), phosphorus (P), potassium (K), and zinc (Zn), and antioxidants (tocopherols, carotenoids, and ascorbic acid) [2,3]. This species was cultivated in 976 ha obtaining a production of 12,152 t in Colombia in 2019 [4]. Likewise, cape gooseberry ranks second in the list of most exported fruits in the country, with Colombia being the first largest producer in the world followed by South Africa [3,5].

Vascular wilt caused by *Fusarium oxysporum* is one of the main limitations in economically important Andean fruit trees such as lulo (*Solanum quitoense* Lamarck.) and cape gooseberry [5,6,7]. This disease caused by *Fusarium oxysporum* f. sp. *physali* (Foph) is the greatest limitation in cape gooseberry production in Colombia. Vascular wilt generates a considerable decrease in production and yield per hectare, going from 19,300 t and 18 t ha^−1^ in 2009 to 16,100 t and 12.2 t ha^−1^ for 2018, respectively [4,5,8].

The fungus is characterized by affecting plants at any phenological stage. The main symptoms of the disease are root rot, chlorosis of the borders and central parts of mature leaves, loss of turgor in young leaves and stems, stunted growth, and finally death of the plant [9,10,11]. The biotic stress by *Fusarium oxysporum* causes different alterations such as decreased nutrient transport and water uptake, reduced plant growth, lower gas exchange and leaf water potential, and increased oxidative stress damage (MDA production) and free amino acid content such as proline [12,13,14,15]. Additionally, the condition generated by *F. oxysporum* is accompanied by oxidative stress [16].

*Fusarium oxysporum* can produce three types of propagules: macroconidia, microconidia, and chlamydospores [6]. In general, microconidia are responsible for the infection of the pathogen in plants, as well as for its dissemination in soil and water [17]. The production of chlamydospores (resistance structures) guarantees the survival of the pathogen under adverse environments and its permanence in the soil for long periods, making its management difficult and causing its control to be limited [18,19].

Vascular wilt management in cape gooseberry has been centered on the application of chemical synthesis fungicides that has caused pathogen resistance to these compounds, environmental pollution, and increased production costs [5,11]. The implementation of cultural practices for the management of this disease, such as the elimination of plants with symptoms of the disease or soil-disinfection methods, are virtually non-existent in the country [11]. The absence of control strategies for this pathogen has generated, in some cases, the total loss of crops [8]. Furthermore, the interest of consumers in acquiring food free of agrochemical residues and the respect for the environment has promoted the creation of alternative management techniques for this type of soil pathogens [11,20].

The use of beneficial microorganisms through the application of biological control agents (BCAs) has become a useful strategy to lessen the impact caused by pathogens [21,22]. BCAs can decrease the impact of fungicides on the environment such as residuality and imbalance in the soil microbiota in production areas [23,24]. Positive results of the use of BCAs have been reported in the management and protection of various crops [23,24]. Likewise, the use of mixtures of different BCAs has generated interest in the possibility to increase efficacy in the management of plant diseases [25,26]. BCA mixtures can increase the effectiveness in disease control by presenting different biocontrol or action mechanisms. Synergistic effects have been also observed because of the complementarity of the action mechanisms between BCAs [26,27].

Some of the species of the genera *Trichoderma* and *Bacillus* are considered of high interest because they have shown biocontrol potential [28,29]. Various action mechanisms such as competition, antibiosis, mycoparasitism, resistance induction, and endophytic activity are reported as biological control in *Trichoderma* species [30,31]. In contrast, the production of antimicrobial substances, competition, colonization, production of lytic enzymes and volatile organic compounds, and induction of plant defenses are reported as action mechanisms in *Bacillus* species [29,32,33]. Furthermore, *Trichoderma* species have been applied with positive results in the management of vascular wilt caused by *F. oxysporum* [34,35]. Similar effects have been observed for the use of *Bacillus* species for vascular wilt control [36,37]. In this regard, previous research studied the biocontrol effect of *Trichoderma virens* and *Bacillus velezensis*, concluding that *T. virens* is promising candidate for the control of vascular wilt in cape gooseberry [38].

Organic additives are also a natural option for disease management and have a biostimulant effect [23]. Within this group of additives, chitosan is considered a biopesticide related to disease management [39,40]. Chitosan has been used as an elicitor of the natural defense response of plants to combat diseases caused by pathogens, as well as presenting mucoadhesive properties [41,42]. Furthermore, this biopolymer has been reported for the control of *F. oxysporum* under different growing and media conditions because of its antimicrobial and biostimulant activities, and its capacity to promote resistance mechanisms in plants [43,44]. Finally, a previous study also showed that chitosan incorporation into the rhizosphere helped the physiology of plants infected with Foph [45].

Organic fertilizers, compost, and burnt rice husk have been considered organic additives because they exert a control effect on soil pathogens [46,47]. Burnt rice husk has shown an effect on the control of different diseases such as: anthracnose (*Colletotrichum* spp.) in tomato [48], root rot (*Cylindrocarpon destructans* and *Fusarium solani*) in ginseng [47], and downy mildew (*Pseudoperonospora* sp.) in bitter gourd (*Momordica charantia* L.) [49].

The combination of BCAs and organic additives may be of interest for disease control in different crops [50,51]. In this sense, the use of the mixture of *Bacillus pumilus* and chitosan in tomato plants inoculated with *F. oxysporum* f. sp. *radicis-lycopersici* obtained promising results in the management of the disease due to increased root resistance to the infection [52].

Plants face many biotic agents (such as viruses, bacteria, fungus, or arthropods), causing biotic stress in their hosts. These agents can disrupt normal metabolism, plant growth, and yield [53]. In Colombia, one of the most limiting biotic agents in cape gooseberry crops is Foph, which is the species that causes vascular wilt, showing a wilt incidence greater than 50% in the production areas [54]. Alternatives based on biological control have become very important for the *P. peruviana*–Foph pathosystem in Colombia [11,38,45]. These studies have allowed selecting promising alternatives and knowing the plant responses to the individual use of BCAs such as *T. virens* or *B. velezensis*. They have also allowed the evaluation of the effect of applications of organic additives such as chitosan or burnt rice husk on vascular wilt management and cape gooseberry plant physiology. However, information on the joint activity of these control tools (BCAs + organic additives) on the disease and its effect on plant physiology remains scarce. Therefore, this research aimed to study the comparative response of the application of three mixtures of BCAs and additives (i) *T. virens* + *B. velezensis* (Mix), (ii) *T. virens* + *B. velezensis* + burnt rice husk (MixRh) or (iii) *T. virens* + *B. velezensis* + chitosan (MixChi) on the plant processes (biochemistry, photosynthetic machinery, water status, and growth) of Foph-inoculated and uninoculated cape gooseberry seedlings.

## 2. Results

### 2.1. Estimation of Vascular Wilt Development by AUDPC, Disease Index and Vascular Browning

**Figure 1:**
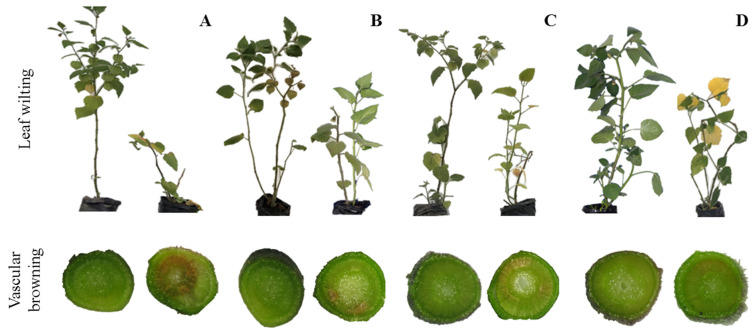
Vascular wilt and vascular browning symptoms in cape gooseberry seedlings infected by *Fusarium oxysporum* f. sp. *physali* (Foph) under treatments with mixtures of biological control agents and organic additives 50 days after inoculation (DAI). The image shows control seedlings (Foph^−^) on the right and pathogen control seedlings (Foph^+^) on the left. (**A**). Symptoms in cape gooseberry seedlings without any treatment. (**B**). Seedlings treated with the mixture *T. virens* + *B. velezensis* (Mix). (**C**). Seedlings with applications of the mixture *T. virens* + *B. velezensis* with the addition of burnt rice husk (MixRh). (**D**). Seedlings with the application of the mixture *T. virens* + *B. velezensis* and the addition of chitosan (MixChi).

Disease incidence in Foph^+^ inoculated plants was 100%. Isolates in PDA medium allowed confirming the pathogen’s presence in symptomatic plants and inoculated with Foph^+^, and its absence was also confirmed in non-inoculated (Foph^−^) cape gooseberry plants (Figure 1). Differences between treatments in the AUDPC (*p* = 0.0058) and disease severity index (*p* = 0.0189) were observed at 50 DAI (Table 1). Cape gooseberry seedlings without the addition of the mixtures and inoculated with Foph^+^ (pathogen control) registered the highest AUDPC values (69.3) (Table 1; Figure 1A). Intermediate values for the AUDPC were observed in plants with the application of the mixture of *T. virens* + *B. velezensis* and the addition of burnt rice husk (MixRh) (63.8) (Table 1; Figure 1C). Finally, the lowest values were recorded for the mixtures *T. virens* + *B. velezensis* (Mix) and *T. virens* + *B. velezensis* with the addition of chitosan (MixChi) (56.6 and 57.6, respectively) (Table 1; Figure 1B–D).

The lowest severity index values were registered in seedlings with the application of the mixture of *T. virens* + *B. velezensis* (Mix) (2.83), while higher severity values were obtained in the pathogen control (Foph^+^) (3.20). Finally, the vascular browning percentage registered similar results to those obtained in the AUDPC. The lowest values of vascular browning were evident in Foph-inoculated plants (Foph^+^) treated with the mixtures *T. virens* + *B. velezensis* (Mix) (3.66) and *T. virens* + *B. velezensis* with the addition of chitosan (MixChi) (4.02) (Table 1). This trend can be observed in Table 1 and Figure 1A, where the greatest values of vascular browning were registered in pathogen control plants (Foph^+^) compared to inoculated seedlings treated with the different mixtures and plants of the absolute control (Foph) (Figure 1B–D).

### 2.2. Growth Parameters

**Figure 2:**
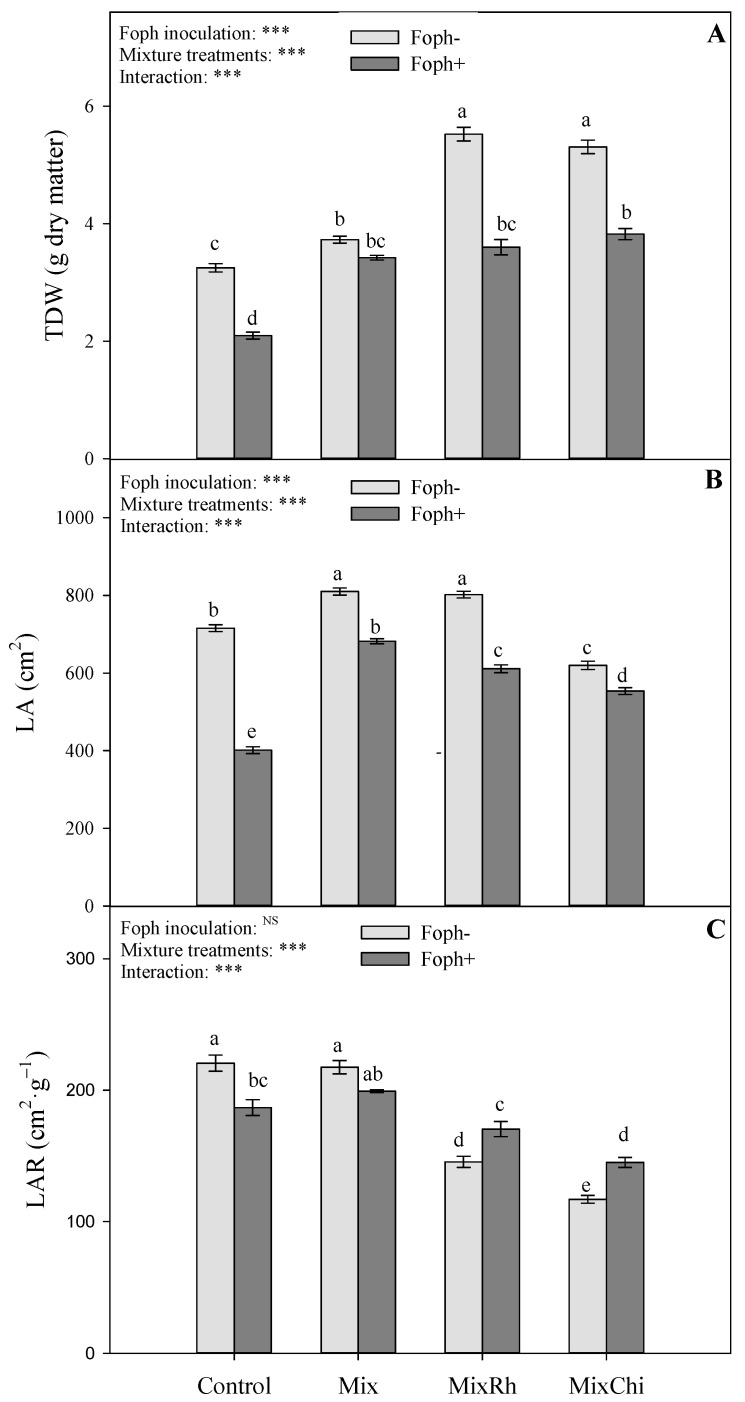
Effect of the application of mixtures of biological control agents and organic additives (Mix (*T. virens* + *B. velezensis*), MixRh (*T. virens* + *B. velezensis* + burnt rice husk), and MixChi (*T. virens* + *B. velezensis* + chitosan)) on (**A**) total dry weight, (**B**) leaf area, and (**C**) leaf area ratio (LAR) of cape gooseberry plants without (light grey bars) and with (dark grey bars) *F.* oxysporum f. sp. *physali* (Foph) inoculation at 50 days after inoculation (DAI). Error bars represent the mean of four values ± standard error. Significant differences between treatments are indicated by different letters according to the Tukey’s test (*p* ≤ 0.05). *** *p* < 0.001values of the ANOVA of Foph inoculation, mixture treatments and their interaction. ^N.S.^ Not significant.

Growth parameters (total dry weight (TDW), leaf area (LA), and leaf area ratio (LAR) of cape gooseberry seedlings displayed differences (*p* = 0.0000, *p* = 0.0000, and *p* = 0.0000, respectively) in the interaction between the presence of Foph and the mixtures (Mix, MixRh, and MixChi) at 50 DAI. The group of plants without pathogen inoculation (Foph^−^) registered the highest growth parameters compared to plants inoculated with the pathogen (Foph^+^) (Figure 2). Regarding TDW, the application of the different mixtures (Mix, MixRh, and MixChi) favored this variable in inoculated (Mix 3.1 g, MixRh 3.6 g, and MixChi 3.8 g) and non-inoculated plants (Mix 3.8 g, MixRh 5.5 g, and MixChi 5.3 g) (Figure 2A). LA was also favored by all mixture treatments in both inoculation situations (with and without Foph), registering the highest values with the application of *T. virens* + *B. velezensis* (Mix) in inoculated (682.1 cm^2^) and non-inoculated (810.2 cm^2^) plants (Figure 2B). Finally, the application of *T. virens* + *B. velezensis* (Mix) also increased (199.2 cm^2^·g^−1^) LAR values mainly in diseased plants compared to the same group of inoculated plants and treated with MixRh and MixChi (~157.7 cm^2^·g^−1^) (Figure 2C).

### 2.3. Stomatal Conductance and Leaf Water Potential

**Figure 3:**
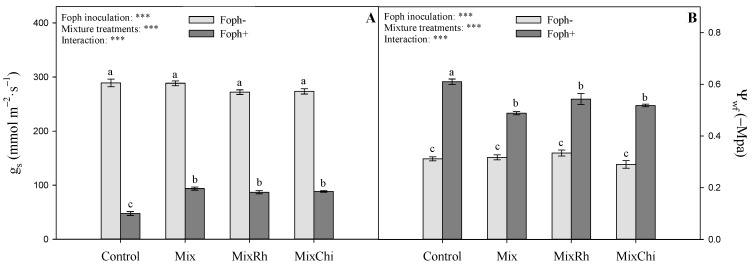
Effect of treatments with mixtures of biological control agents and organic additives (Mix (*T. virens* + *B. velezensis*), MixRh (*T. virens* + *B. velezensis* + burnt rice husk) and MixChi (*T. virens* + *B. velezensis* + chitosan) on (**A**) stomatal conductance (g_s_) and (**B**) leaf water potential (Ψ*_wf_*) of cape gooseberry seedlings without (light grey bars) and with (dark grey bars) *F. oxysporum* f. sp. *physali* (Foph) inoculation at 50 days after inoculation (DAI). Error bars represent the mean of four values ± standard error. Significant differences between treatments are indicated by different letters according to the Tukey’s test (*p* ≤ 0.05). *** *p* < 0.001—values of the ANOVA of Foph inoculation, mixture treatments, and their interaction.

The stomatal conductance (g_s_) and leaf water potential (Ψ*_wf_*) are summarized in Figure 3. Differences were also observed between Foph inoculation and treatments with mixtures on g_s_ (*p* = 0.0000) and Ψ*_wf_* (*p* = 0.0000) at 50 DAI. In general, cape gooseberry plants with Foph^+^ showed lower g_s_ compared to non-inoculated plants (~79.2 mmol m^−2^ s^−1^ and ~280.7 mmol m^−2^ s^−1^, respectively). However, the application of treatments with the different mixtures (Mix, MixRh, and MixChi) favored g_s_ compared to inoculated plants (Foph^+^) without application of mixtures (~90 mmol m^−2^ s^−1^ and 47.6 mmol m^−2^ s^−1^, respectively) (Figure 3A). The Ψ*_wf_* displayed similar trends to those recorded for g_s_. The group of non-inoculated plants (Foph^−^) showed a higher water status (Ψ*_wf_* ~ −0.32 Mpa) compared to Foph^+^ plants (Ψ*_wf_* ~ −0.53 Mpa). However, the application of different mixtures favored Ψ*_wf_* in Foph^+^ inoculated plants by 17% (Figure 3B).

### 2.4. Chlorophyll and Carotenoid Content

**Figure 4:**
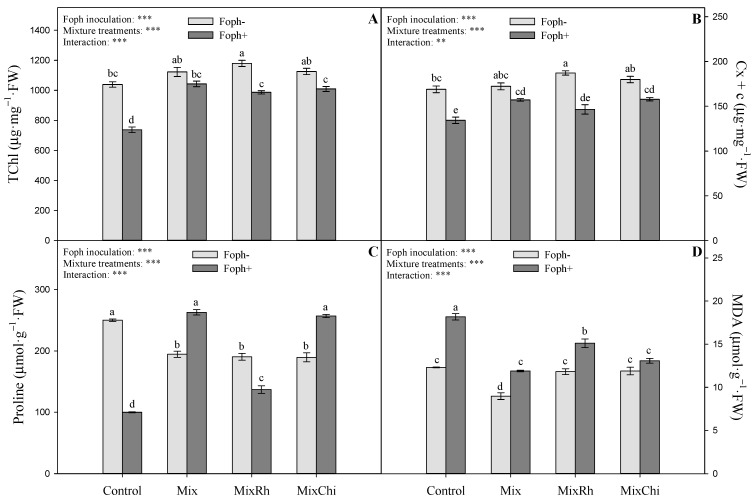
Effect of treatments with mixtures of biological control agents and organic additives (Mix (*T. virens* + *B. velezensis*), MixRh (*T. virens* + *B. velezensis* + burnt rice husk), and MixChi (*T. virens* + *B. velezensis* + chitosan) on the contents of (**A**) total chlorophyll (TChl) and (**B**) carotenoids (Cx + c), (**C**) proline and (**D**) malondialdehyde (MDA) of cape gooseberry plants without (light grey bars) and with (dark grey bars) of *F. oxysporum* f. sp. *physali* (Foph) inoculation at 50 days after inoculation. Error bars represent the mean of four values ± standard error. Significant differences between treatments are indicated by different letters according to the Tukey’s test (*p* ≤ 0.05). ** *p* < 0.01 and *** *p* < 0.001—values of the ANOVA of Foph inoculation, mixture treatments and their interaction.

Total chlorophyll (TChl) and carotenoid (Cx + c) contents are shown in Figure 4A,B. The evaluated factors (presence of Foph x mixtures) showed differences (*p* = 0.0000) in the photosynthetic pigment content at the end of the experiment (50 DAI). Chlorophyll and carotenoid contents were lower in plants inoculated with the pathogen (Foph^+^) compared to plants without pathogen inoculation (Foph^−^) (Figure 4A,B). It was observed that the concentration of TChl was favored by the application of the different mixtures (Mix, MixRh, and MixChi) in Foph-inoculated cape gooseberry plants, registering average values of 1012.8 µg^−1^ mg fresh weight (FW) compared to pathogen control plants (Foph^+^) (737.3 µg^−1^ mg FW). The application of the mixture *T. virens* + *B. velezensis* (Mix) in plants without Foph presence recorded the highest TChl values (Figure 4A). The lowest values of Cx + c content was registered in pathogen control plants (Foph^+^) (134.31 µg^−1^ mg PF). An increase in the values of this photosynthetic pigment was also observed with the application of the Mix and MixChi treatments in cape gooseberry plants with Foph^+^ (157.1 and 157.8 µg^−1^ mg FW). The highest Cx + c values were recorded in Foph-plants treated with MixRh (Figure 4B).

### 2.5. Malondialdehyde and Proline Content

Differences (*p* = 0.0000) were recorded between inoculation with Foph and the treatment with mixtures for the variable’s proline content and lipid membrane peroxidation expressed as MDA content at 50 DAI (Figure 4C,D). The lowest levels of proline content were registered in pathogen control plants (Foph^+^) (99.91 µmol g^−2^ FW). The use of the different mixtures (Mix, MixRh, and MixChi) promoted proline synthesis, with the mixtures *T. virens* + *B. velezensis* (Mix) and *T. virens* + *B. velezensis* with the addition of chitosan (MixChi) being those that generated the greatest proline accumulation (262.86 µmol g^−2^ FW and 256.86 µmol g^−2^ FW, respectively) (Figure 4C). In contrast, lower MDA values were recorded with the application of the different mixtures, mainly with *T. virens* + *B. velezensis* (Mix) (11.9 µmol g^−2^ FW) and *T. virens* + *B. velezensis* with the addition of chitosan (MixChi) (13.1 µmol g^−2^ FW) compared to pathogen control plants (Foph^+^) (18.2 µmol g^−2^ FW) (Figure 4D). The lowest values of lipid peroxidation were observed in Foph^−^ seedlings, especially with the Mix treatment (8.9 µmol g^−2^ FW) (Figure 4D).

### 2.6. Efficacy of Mixtures of Biological Control Agents (BCAs) and Organic Additives and Relative Tolerance Index (RTI)

**Figure 5:**
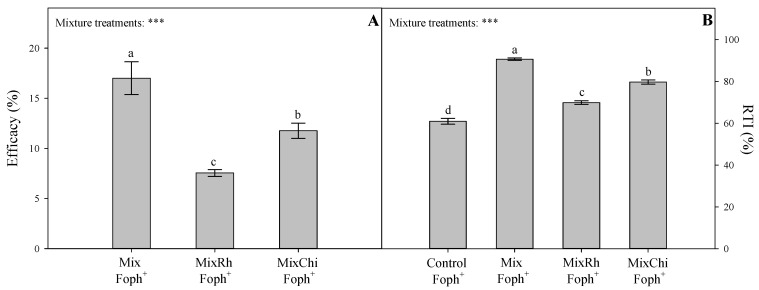
Effect of the application of mixtures of biological control agents and organic additives (Mix (*T. virens* + *B. velezensis*), MixRh (*T. virens* + *B. velezensis* + burnt rice husk), and MixChi (*T. virens* + *B. velezensis* + chitosan) on (**A**) the efficacy of control and (**B**) relative tolerance index (RTI) of cape gooseberry seedlings infected by *F. oxysporum* f. sp. *physali* (Foph) at 50 days after inoculation (DAI). Bars represent the mean of four values ± standard error. Significant differences between treatments are indicated by different letters according to the Tukey’s test (*p* ≤ 0.05). *** *p* < 0.001—values of the ANOVA of mixture treatments are indicated as.

The highest values of percentage of efficacy were recorded in plants treated with the mixtures *T. virens* + *B. velezensis* (Mix) and *T. virens* + *B. velezensis* with the addition of chitosan (MixChi) (17% and 11.8%, respectively) (Figure 5A). The previous analysis is also confirmed by Figure 1 where pathogen control plants (Foph^+^) showed the greatest symptoms of vascular wilt compared to plants of the different treatments with the mixtures and absolute control plants (Foph).

The relative tolerance index (RTI) based on total dry weight (TDW) validated previous observations of disease monitoring and the evaluated physiological variables. The applications of the different mixtures (Mix, MixRh, and MixChi) helped plants to tolerate the biotic stress condition caused by Foph infection. The highest values of RTI were observed in inoculated plants with the application of the mixture *T. virens* + *B. velezensis* (Mix) (90.6%), followed by the mixtures *T. virens* + *B. velezensis* with the addition of chitosan (MixChi) (79.7%) and *T. virens* + *B. velezensis* with the addition of burnt rice husk (MixRh) (69.9%). Plants inoculated with the pathogen (Foph^+^) showed the lowest RTI value (60.9%) (Figure 5B).

### 2.7. Correlation between Physiological Parameters, Disease Monitoring, and RTI

**Figure 6:**
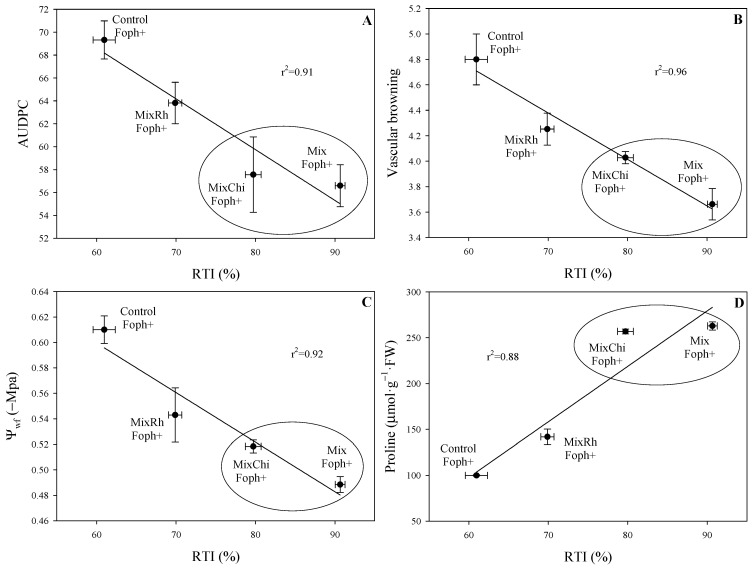
Correlation between (**A**) the area under the disease progress curve (AUDPC), (**B**) vascular browning, (**C**) leaf water potential (Ψ*_wf_*), or (**D**) proline content and the relative tolerance index (RTI) in cape gooseberry seedlings infected with *F. oxysporum* f. sp. *physali* (Foph) and treated with the application of mixtures of biological control agents and organic additives (Mix (*T. virens* + *B. velezensis*), MixRh (*T. virens* + *B. velezensis* + burnt rice husk), and MixChi (*T. virens* + *B. velezensis* + chitosan) at 50 days after inoculation (DAI). Each point shows the average of five plants. Vertical and horizontal bars represent ± standard error per treatment (*n* = 5). Circles represent the group of treatments with greater tolerance to Foph inoculation.

AUDPC (*p* = 0.006) and vascular browning (*p* = 0.0033) showed a high negative correlation (r^2^ = 0.91 and 0.96, respectively) with RTI. These correlations also showed that treatments with the application of mixtures *T. virens* + *B. velezensis* (Mix) and *T. virens* + *B. velezensis* with the addition of chitosan (MixChi) showed the lowest AUDPC and vascular browning values with the highest RTI values (Figure 6A,B). On the other hand, leaf water potential (Ψ*_wf_*) (*p* = 0. 0.0052) and proline content (*p* = 0.0147) showed a high positive correlation (r^2^ = 0.92 and 0.88, respectively), registering high Ψ*_wf_* and proline values in the treatments *T. virens* + *B. velezensis* (Mix) and *T. virens* + *B. velezensis* with the addition of chitosan (MixChi) with the highest RTI values (Figure 6C,D).

### 2.8. Comparative Analysis of Vascular Wilt Mitigation by the Application of Mixtures of Biological Control Agents (BCAs) and Organic Additives

**Figure 7:**
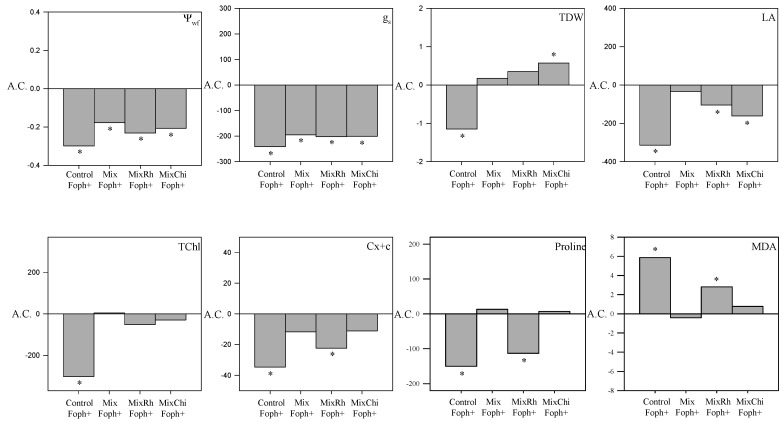
Comparative effect of the variables evaluated in cape goose berry plants inoculated and treated with mixtures of biological control agents and organic additives (Mix (*T. virens* + *B. velezensis*), MixRh (*T. virens* + *B. velezensis* + burnt rice husk), and MixChi (*T. virens* + *B. velezensis* + chitosan) in relation to the behavior of absolute control plants (Foph) 50 days after inoculation. A.C. indicates the average value of absolute control plants for each of the parameters. * indicates the statistical differences in the treatment compared to control plants (Foph).

**Figure 8:**
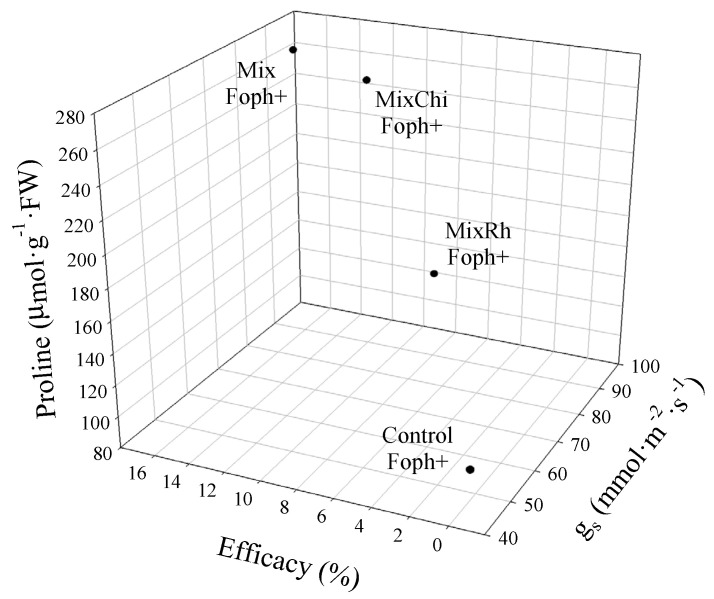
Three-dimensional analysis among stomatal conductance (g_s_), percentage of efficacy, and proline content to identify the best treatments of mixtures of biological control agents and organic additives (Mix (*T. virens* + *B. velezensis*), MixRh (*T. virens* + *B. velezensis* + burnt rice husk), and MixChi (*T. virens* + *B. velezensis* + chitosan) on the control and physiological behavior of cape gooseberry seedlings inoculated with *F. oxysporum* f. sp. *physali* (Foph^+^).

Correlations between some disease monitoring and physiological variables (AUDPC, vascular browning, Ψ*_wf,_* and proline content) and RTI showed that treatments with the mixtures *T. virens* + *B. velezensis* (Mix) and *T virens* + *B. velezensis* with the addition of chitosan (MixChi) mitigated the negative effect generated by the inoculation of the pathogen. These treatments (Mix, MixChi, and pathogen control (Foph^+^)) were compared to the group of seedlings without inoculation of the pathogen (Foph^−^) and without any mixture treatment (absolute control) at 50 DAI. These results showed that applications of *T. virens* + *B. velezensis* (Mix) and *T. virens* + *B. velezensis* with the addition of chitosan (MixChi) helped plants to cope with the Foph inoculation condition since a positive effect of these mixtures was observed on g_s_, Ψ*_wf_*, TDW, LA, TChl, Cx + c, MDA, and proline content (Figure 7). The three-dimensional graph (percentage of efficacy, proline, and g_s_) confirmed the correlations and comparative analysis described above. The three-dimensional analysis among physiological, biochemical, and disease monitoring variables showed that applications of mixtures of BCAs or BCAs + organic additives (Mix or MixChi) can decrease the levels of vascular wilt and could be considered for both the response against pathogens or the mitigation of stress conditions by promoting plant physiological processes (Figure 8).

## 3. Discussion

Positive effects on the management of vascular wilt have been reported separately for the application of BCAs (*Trichoderma* or *Bacillus*) [26,36,55] and organic additives such as chitosan [46,56]. However, recent reports on the use of these compounds in mixtures for the management of vascular wilt are still scarce [52,57,58]. In this study, the different mixtures (Mix, MixRh, or MixChi) exerted control over the disease. This was mainly observed for treatments with *T. virens* + *B. velezensis* (Mix) or *T. virens* + *B. velezensis* with the addition of chitosan (MixChi), which showed lower AUDPC, severity index, and vascular browning values in cape gooseberry seedlings (Table 1; Figure 1). Izquierdo-García et al. [26] also observed that the use of a mixture of *T. virens* and *B. velezensis* lowered vascular wilt (Foph) severity in cape gooseberry. Bakeer et al. [59] similarly observed that the joint application of chitosan with *T. harzianum* and *B. suptilis* reduced *Fusarium* wilt severity in tomato (*Solanum lycopersicum* L.) plants by 71% compared to plants without treatments. The application of combinations of BCAs and organic additives can also benefit plants by promoting better growth and development, greater association with the soil microbial community, and higher effectiveness in the control of pathogens [60].

*Fusarium oxysporum* infection causes direct negative effects such as lower the leaf gas exchange properties, dry matter, plant water status, and photosynthetic pigments, and a higher MDA and proline production [12,13,14,15]. This research showed that the applications of mixtures of BCAs and organic additives (Mix, MixRh, or MixChi) helped plants to cope with the negative effects caused by Foph on their physiological and biochemical responses. The variables g_s_, Ψ*_wf_*, TDW, LA, photosynthetic pigments, and MDA and proline contents were positively affected by the treatments mainly with *T. virens* + *B. velezensis* (Mix) or *T. virens* + *B. velezensis* with the addition of chitosan (MixChi). Okorski et al. [61] obtained similar responses with the application of a mixture of different BCAs (biological preparation of effective microorganisms such as lactic acid and photosynthetic bacteria and yeast), which also generated an increase in gas exchange parameters such as photosynthesis, g_s,_ and transpiration in pea plants (*Pisum sativum* L.) with *Fusarium oxysporum* wilt symptoms. A previous study also indicated that Foph-infected cape gooseberry plants and treated with chitosan showed better gas exchange (g_s_) and Ψ*_wf_* parameters compared to plants without chitosan [45].

Vascular wilt also causes changes in the distribution of plant assimilates and reduces water and nutrient transport which is reflected in lower growth [62]. However, in the present study growth (TDW, LA, and LAR) was favored in plants inoculated and treated with the different mixtures, with similar or superior behavior than that of absolute control plants (Foph^−^) (Figure 2 and Figure 7). Zaim et al. [63] also recorded an increase in plant height, root length, and fresh and dry matter of shoots and roots in chickpea plants inoculated with *F. oxysporum* f. sp. *ciceris* with the joint application of BCAs *B. subtilis* and T. *harzianum*. Likewise, applications of chitosan in mixture with *B. subtilis* and *T. harzianum* have shown higher crop yield of 61% compared to diseased tomato plants without the application of these mixtures [59].

The contents of MDA, proline, and leaf photosynthetic pigments can be used as biochemical markers to quantify the plant’s response to biotic stress conditions [64,65,66]. Treatments mainly with Mix and MixChi favored the concentration of photosynthetic pigments (TChl and Cx + c) and proline, and decreased MDA accumulation in Foph-infected plants (Figure 4 and Figure 7) in this study. A higher concentration of photosynthetic pigments (TChl and Cx + c) and proline has also been reported in lettuce (*Lactuca sativa* L.) plants infected with *Rhizoctonia solani* and with the application of a mixture of two bio fungicides formulated with *T. harzianum* and *B. subtilis* [67]. On the other hand, treatments with the mixture of three plant growth-promoting rhizobacteria (PGPR) of the genus *Pseudomonas* and chitosan significantly increased (>65%) chlorophyll content (SPAD) in tomato plants infected with tomato leaf curl virus (ToLCV) [68]. Likewise, Zhang et al. [69] registered a decrease in MDA production after treatment with the mixture of chitosan and the antagonistic yeast *Rhodotorula mucilaginosa* in strawberry (*Fragaria ananassa* Duch.) plants inoculated with *Rhizopus stolonifer* and *Botrytis cinerea*.

In this study, it was also found that treatments with mixtures of BCAs and organic additives such as chitosan helped to ameliorate the effects caused by Foph through the improvement of the water potential, stomatal behavior, and biochemical expression and the decrease of vascular wilt. This response may be caused by the fact that BCAs such as *T. virens* may participate in the activation of single or multiple biocontrol mechanisms against plant diseases, including the production of hydrolytic enzymes such as β-1,3-glucanases, chitinases and proteases (mycoparasitism), segregation of iron-chelating siderophores to suppress pathogen growth (competition), and production of secondary metabolites for resistance induction [22,70,71]. *B. velezensis* can contribute to the antagonistic action against pathogens through antibiosis and direct competition for the secretion of different secondary metabolites with antibacterial and antifungal activity (lipopeptides) in the rhizosphere. It can also benefit the host plant microbiome and stimulate induced systemic resistance (ISR) mediated by the production of elicitors such as jasmonic and ethylene salicylic acids [72,73,74].

The beneficial effect of BCAs on the water status and gas exchange properties (g_s_) of plants could be related to a greater and better mineral availability in the soil. This may improve nutrient uptake and movement in plants, the efficient use of water, and the overexpression of proteins such as aquaporins that improve water and solute transport [61,75,76]. BCAs can synthesize growth hormones such as indole-3-acetic acid and gibberellic acid that promote plant growth and increased nutrient uptake through the production of secondary metabolites [77,78]. Additionally, the application of BCAs can regulate the biosynthesis of proteins and chlorophyll in plants (activation of porphobilinogen synthase enzyme) [79]. Finally, the positive results of BCAs application could be associated with increased activity of antioxidant enzymes (catalase, superoxide dismutase, and ascorbate peroxidase), and the induction of proline metabolism, which decreases the levels of lipid peroxidation of membranes [80,81].

Chitosan treatments decreased vascular wilt severity in cape gooseberry plants since they may play a role in the induction of plant defense, the activation of enzymes such as chitinases and β-1,3-glucanase, the biosynthesis of phytoalexin, the generation of reactive oxygen species, and the synthesis of inhibitors of callose and protease that affect fungal growth [39,82]. Furthermore, this biopolymer shows elicitor activity through antimicrobial activity (production secondary metabolites such as phenolic compounds), induction of systemically acquired plant resistance against a wide range of pathogens [42], and has mucoadhesive properties that improve permeation and can prolong the positive effects of compounds with chitosan [41]. Additionally, the use of chitosan generated an increase in g_s_, Ψ*_wf,_* and growth of cape gooseberry plants, probably due to the promotion of root development, the increase in water and nutrient uptake, the stimulation of osmotic adjustment which facilitates the accumulation of compatible solutes, and the regulation of processes such as elongation and division of cells, activation of enzymes, and synthesis of proteins under stress conditions [83,84,85]. Finally, chitosan treatments also favored the biochemical behavior (photosynthetic pigments, MDA, and proline) of diseased cape gooseberry plants. These responses could be related to the protection of the photosynthetic complex from protein and lipid oxidative damage in the chloroplast [86], the reduction of oxidative stress caused by chitosan’s ability to bind with proteins and macromolecules, metal ions and negatively charged lipids, and the induction of free amino acid accumulation (proline) related to the osmotic adjustment and antioxidant defenses of stressed plants [87].

The use of burnt rice husk in the mixture with BCAs had a lower effect on vascular wilt control and the physiological and biochemical responses of cape gooseberry plants compared to the Mix and MixChi treatments (Table 1; Figure 1, Figure 2, Figure 3, Figure 4, Figure 5, Figure 6, Figure 7 and Figure 8). Araujo et al. [88] showed that the application of a mixture of charcoal (biochar) and *T. harzianum* inhibited mycelial growth of *Macrophomina phaseolina* and stimulated the germination percentage, number of pods, and dry and fresh matter of bean (*Phaseolus vulgaris* L.) plants. The positive response to the application of this type of compounds (charcoal) may be related to the promotion of beneficial microorganisms’ growth, the improvement of nutrient solubilization and uptake, the neutralization of phytotoxic compounds in the soil and the induction of plant defense mechanisms [89,90].

It is convenient to indicate that the external spores in the parts that remain above ground level (field conditions) are dispersed by the wind, water, people and equipment, and by the movement of soil particles that contain the fungus, hence the importance and benefit of considering the presence of *Fusarium* wilt in commercially important crops and favorable environmental conditions such as soil, climate, management, agronomic, among others [91]. Our results could serve as a basis for future lines of research by pioneers in Plant Protection sciences and biotic interactions in Colombia and others countries, where *Fusarium* wilt has been a big issue in crop protection of different crops such as banana (TR4) [92,93]. These findings also be a fundamental contribution to avoid the spread and devastation of plantations of commercially important crops such as Cape gooseberry.

## 4. Materials and Methods

### 4.1. Microorganisms and Culture Conditions

Strain Map5 of *F. oxysporum* f. sp. *physali* (Foph) and the BCAs *Trichoderma virens* and *Bacillus velezensis* were provided by the Microorganisms Collection of Corporación Colombiana de Investigación Agropecuaria—AGROSAVIA. Foph inoculum at an initial concentration of 1 × 10^6^ microconidia·mL^−1^ was grown for 7 days on sterile potato-dextrose broth (PDB, Difco^®^) under continuous agitation (125 rpm) at 25 °C. The fermented broth was then filtered using three layers of sterile muslin cloth and centrifuged at 15,000 rpm for 15 min. The obtained biomass was rinsed twice with sterile distilled water (SDW). The microconidia obtained were re-suspended in SDW, adjusting the suspension at 1 × 10^6^ microconidia·mL^−1^ using a Neubauer chamber for counting. *T. virens* was grown for seven days on potato-dextrose-agar (PDA) and conidia were harvested by scraping with SDW to obtain the inoculum, which was also adjusted by Neubauer chamber count to 1x10^6^ microconidia·mL^−1^. *B.* *velezensis* was grown in Luria Bertani broth (LB, Tryptone 10 g, NaCl 10 g and yeast extract 5 g·L^−1^) at 25 °C using an orbital shaker at 125 rpm for continuous agitation for 48 h. The bacterial suspension concentration was adjusted by using a spectrophotometer (BIOTEK^®^, Winooski, VT, USA) to measure the optical density (OD_600 nm_= 1 × 10^8^ cells. mL^−1^).

### 4.2. Plant Material and Growth Conditions

An experiment was carried out in the greenhouses of the Faculty of Agricultural Sciences of the Universidad Nacional de Colombia, Bogotá campus (4°35′56″ N, 74°04′51″ W, altitude 2557 m) between February and June 2017. The climatic conditions during the study were as follows: a natural photoperiod of 12 h (photosynthetically active radiation (PAR) 1500 µm^−1^s^−2^ at noon), day/night temperature of 25/20 °C, and relative humidity of ~72%. Commercial seeds (Semicol S.A., Bogotá, Colombia) of cape gooseberry ecotype ‘Colombia’ (highly susceptible to vascular wilt) [19,38], were subjected to superficial disinfection by immersion in a 70% ethanol solution (*v*/*v*) for 1 min, 3% sodium hypochlorite (*v*/*v*) for 20 min with agitation, and three washes using sterile distilled water. Additionally, the seeds destined for chitosan application were immersed in a chitosan solution (0.1% p/v) with constant agitation at 150 rpm for 20 min.

After disinfection, seeds sown in 70-cell germination trays using nutrient-free peat (Klasmann^®^, Klasmann-Deilmann GmbH, Germany) as substrate. Thirty days after sowing (DAS) (seed germination), 10 mL of liquid compound fertilizer (N, P, K, and micronutrients) (Nutriponic^®^, Walco S.A., Bogotá, Colombia) was used to irrigate seedlings at a concentration of 3 mL per liter of water every 3 days until transplantation (45 DAS). When four fully expanded leaves were observed in the seedlings, they were transplanted into 2 L plastic pots containing the appropriate substrate based on the treatment and Foph presence or absence.

### 4.3. Treatments with or without Foph Inoculation and the Addition of Mixtures of Biological Control Agents (BCAs) and Organic Additives

The microorganisms selected for the mixtures were *Trichoderma virens* and *Bacillus velezensis* (formerly *B. amyloliquefaciens*) due to their biocontrol potential against plant pathogens [11,38]. Regarding organic additives, chitosan (Sigma Aldrich, St. Louis, MO, USA) and burnt rice husk were also selected due to their potential control of Foph observed in a previous study [45]. Eight groups of treatments were obtained for the development of the experiment: (i) cape gooseberry plants with no addition of BCAs or organic additives and inoculated with Foph (pathogenic control (Foph^+^)) or without it (absolute control (Foph^−^)); (ii) cape gooseberry plants with or without Foph inoculation and with the application of the mixture of BCAs (*T. virens* and *B. velezensis* (Mix)); (iii) cape gooseberry plants with or without Foph inoculation with the application of the mixture of BCAs and the addition of chitosan (MixChi) and (iv) cape gooseberry plants with or without Foph inoculation with application of the mixture of BCAs and the addition of burnt rice husk (MixRh). Plants of treatments 1, 2, and 3 were established in substrate that contained a mixture of soil and rice husk at a 3:1 ratio (*v*/*v*), whereas burnt rice husk was incorporated into the soil at the time of transplantation obtaining a soil-husk substrate at a 3:1 ratio (*v*/*v*) in treatment 4. These substrate mixtures have been used in previous studies in which the individual effect of BCAs or organic additives on vascular wilt in cape gooseberry plants was compared [38,45]. Finally, the pathogen’s absence in the soil used in the preparation of substrate mixtures was confirmed previously by the technique described by Park [94], by adding 100 mL of cool-molten galactose-nitrate agar (GNA) medium with 10 µg·mL^−1^ benomyl and 300 µg·mL^−1^ chloramphenicol to the soil sample. *F. oxysporum* colonies were counted after incubation for 7 days at room temperature.

The treatments with BCAs were carried out using a mixture of the suspensions of *T. virens* (1 × 10^6^ conidia mL^−1^) and *B. velezensis* (1 × 10^8^ cells mL^−1^) in sterile distilled water (SDW). For this, the Petri dish was scrapped to harvest *T. virens* conidia, and the PDA medium on which the fungus grew was liquefied in Ultra-Turrax^®^ adding 15 mL of SDW per Petri dish to obtain the fungus supernatant. The suspension was centrifuged (15,000 rpm, 15 min, 4 °C) and the obtained supernatant was filtered by 0.22 μm filters (Sartorius^®^). The fermented broth of *B. velezensis* was centrifuged (under the conditions described before) to separate the biomass from the supernatant. The supernatant was harvested and filtered using 0.22 μm filters and *B. velezensis* biomass was rinsed twice with SDW to remove any residues of supernatant [26]. Five milliliters of the combined suspension was applied in drench to each cell in the germination trays (30 DAS), and 30 mL was applied to each of the pots at the time of transplantation (at 45 DAS) for Mix and MixRh treatments, respectively. The mixture of BCAs and chitosan (MixChi) was performed as follows: (i) a first application of chitosan was carried out at seed disinfection, (ii) then, BCAs were drench-applied using 5 mL of the combined suspension (*T. virens* and *B. velezensis*) in germination trays (30 DAS), and (iii) a combined application of BCAs (15 mL) and chitosan (15 mL) was performed at the time of transplantation (45 DAS). The concentration used for chitosan applications was 0.1% (*w*/*v*) at both moments.

*Fusarium oxysporum* f. sp. *physali* inoculation was performed at transplantation by incorporating propagules (microconidia) into the substrate [5]. In this regard, for each 1.0 kg of substrate used, 100 mL of SDW was added with or without the presence of *F. oxysporum* f. sp. *physali* strain Map5 (highly virulent) microconidia [11]. Eight treatment groups were arranged in a completely randomized design with each treatment consisting of eight plants (replicates). Finally, the experiment lasted 95 days.

### 4.4. Disease Severity Analysis

The disease was evaluated by visual inspection of plants using the six-level scale created by Moreno-Velandia [95]; this scale considered the characteristic symptoms of the disease (epinastic response, chlorosis, turgor loss in leaves and plant defoliation until total wilting). For each of the treatments, disease severity was determined every 3 days after inoculation (45 DAS) until the end of the trial. Equation (1) proposed by Chiang et al. [96] was used to calculate the severity index.

(1) Disease severity index=(∑(nv)/V)

where *n* represents the level of affectation based on the scale, *v* is the number of plants present at each level, and *V* is the total number of assessed plants.

The area under the disease progress curve (AUDPC) was determined in each treatment to obtain the severity of the disease using the trapezoidal integration method [97]:

(2) $$\mathrm{AUDPC}=\left\{\sum_{i\;=1}^{n-1}\left[\left(y_{i}+y_{i+1}\right)/2\right]*\left(t_{i+1}-t_{i}\right)\right\}$$

where *n* is the number of assessments, $y_{i\;}$ and $y_{i+1}$ are the values of the severity scale obtained at each evaluation moment, and ($t_{i+1}-t_{i}$) is the time between assessments. Isolates on Potato Dextrose Agar (PDA) medium from explants collected from the stem base confirmed the pathogen’s presence or absence in Foph^+^ inoculated or non-inoculated plants (Foph) [98].

Vascular browning was evaluated 50 days after inoculation (DAI) in cross-sections of the stem base in each treatment. The vascular browning percentage was quantified using a five-level scale proposed by Mandal et al. [99], where 1 = no vascular browning; 2 = 1–25% of vascular browning; 3 = 26–50% of vascular browning; 4 = 51–75% of vascular browning; 5 = more than 75% of vascular browning.

Finally, the efficacy of each of the treatments was calculated using the formula described by Abbott [100] with some modifications Equation (3):

(3) $$Efficacy\;\left(\&\right)=\frac{\left(X-Y\right)}{X}\times 100$$

where *X* represents the severity index of the pathogen control (Foph^+^) and *Y* is the severity index of each treatment at the end of the experiment.

### 4.5. Stomatal Conductance and Leaf Water Potential

Stomatal conductance (g_s_) and leaf water potential (Ψ*_wf_*) were determined using a fully expanded leaf randomly taken from the upper or middle section of the plant’s canopy. g_s_ was estimated with a steady-state porometer (SC-1, Decagon Devices Inc., Pullman, WA, USA). Subsequently, Ψ*_wf_* was estimated using a Schollander pressure chamber (PMS, Model 615, OR) considering the same leaf used to determine g_s_ on totally sunny days at 50 DAI between 9:00 and 12:00 h.

### 4.6. Growth Parameters

The different organs (leaves, stems, and roots) of each plant per treatment were gathered at 50 DAI to obtain their dry weight. Leaf area was determined using digital images in TIFF format (Tagged Image File Format) (D3300, Nikon, Thailand); the images were analyzed using a Java image processing program (Image J; National Institute of Mental Health, Bethesda, MD, USA). The leaf–area ratio was obtained using the ratio between leaf area and total dry weight (TDW) as an indicator of biomass partitioning. Finally, the relative tolerance index (RTI) was also estimated using the TDW and calculated with Equation (4) described by Roussos et al. [101].

(4) $$RTI=\left(\frac{\mathrm{Total}\;\mathrm{biomass}\;\mathrm{of}\;\mathrm{inoculated}\;\mathrm{plants}\;}{\mathrm{Total}\;\mathrm{biomass}\;\mathrm{of}\;\mathrm{plants}\;\mathrm{without}\;\mathrm{inoculation}}\right)\times \;100$$

### 4.7. Chlorophyll and Carotenoid Content

Leaves from the middle third of each treatment were used to take a 0.03 g sample at 50 DAI. Then, liquid nitrogen was used to macerate the leaves, which were then homogenized in 4 mL of 80% acetone. To remove particles, the samples were then centrifuged (Model 420101, Becton Dickinson Primary Care Diagnostics, MD, USA) at 5000 rpm for 10 min. We added acetone to the supernatant to complete a final volume of 6 mL. Finally, spectrophotometer readings (Spectronic BioMate 3 UV-vis Thermo, Madison, WI, USA) were performed at wavelengths of 663 and 646 nm for chlorophyll and 470 nm for carotenoids. The equations proposed by Lichtenthaler [102] were considered to determine the content of these pigments.

### 4.8. Malondialdehyde and Proline Content

The thiobarbituric acid method [103] was used to determine lipid oxidation (Malondialdehyde—MDA). At 50 DAI, 0.3 g of leaves from the upper or middle section of plants from each treatment were macerated and stored in liquid nitrogen. The samples were then centrifuged at 5000 rpm for 10 min and the absorbances were determined at 440, 532, and 600 nm using a spectrophotometer. MDA concentration was obtained using the extinction coefficient (157 M mL^−1^).

Leaf proline concentration was determined using the ninhydrin acid method [104]. Leaf samples of 0.3 g from leaves from the upper or middle section of the canopy of all treatments were also macerated in liquid nitrogen. Then, 10 mL of a 3% sulfosalicylic acid aqueous solution was added. Subsequently, samples were filtered using no. 2 Whatman paper; 2 mL of this filtrate was reacted with 2 mL of ninhydrin acid and 2 mL of glacial acetic acid. The mixture was left in a water bath at 90 °C for 1 h, stopping the reaction by incubation in ice. Four milliliters of toluene were used to dissolve the resulting solution which was shaken with a vortex shaker (V-1, BOECO, Hamburg, Germany) for 30 s. Finally, the absorbance was determined at 520 nm using a spectrophotometer. A standard calibration curve (Equation (5)) was used to determine the proline content with the fresh weight of the sample.

(5) $$\frac{\mu mol\;proline}{g\;fresh\;plant\;material}=\frac{\left[\frac{\frac{\mu g\;proline}{mL}\times mL\;Toluene}{\frac{115.5\;\mu g}{\mu mol}}\right]}{\left[\frac{sample\;g}{5}\right]}\;\;$$

### 4.9. Experimental Design and Data Analysis

A factorial design was used for the data analysis, in which the first factor corresponded to the inoculation (with and without Foph) and the treatments used (Mix, MixRh, MixChi, and control) as a second factor. Each of the treatments consisted of eight plants per replicate. An analysis of variance (ANOVA) was performed, and a Tukey’s post hoc test was used for comparison of means when significant differences (*p* ≤ 0.05) were found. A correlation analysis between RTI and AUDPC, vascular browning, Ψ*_wf,_* or proline was carried out to obtain the best treatments under inoculation conditions (Foph^+^). Additionally, the comparison of the treatment effect on the evaluated variables was made taking as reference the response of the normalized absolute control. The arcsine function was used to transform percentage values. Statistix v 9.0 (Analytical Software, Tallahassee, FL, USA) was utilized to analyze data and SigmaPlot (version 12.0; Systat Software, San José, CA, USA) was used to make the figures, a three-dimensional graph, and perform the correlation analysis.

## 5. Conclusions

In summary, the results of this research indicated that the use of the different mixtures had a positive impact on cape gooseberry plants to mitigate vascular wilt. However, the mixtures *T. virens* + *B. velezensis* (Mix) and *T. virens* + *B. velezensis* with the addition of chitosan (MixChi) obtained the highest efficiencies in controlling the disease. Additionally, these treatments (Mix or MixChi) showed a biostimulant and growth-promoting effect, lower damage to membranes (low MDA contents), and pigment contents similar to those obtained in absolute control plants (Foph^−^). These results suggest that the use of these mixtures in cape gooseberry plants could be considered as a complementary tool for the integrated management of the disease in areas where this crop is produced.

## Figures and Tables

**Table 1:** Area under the disease progress curve (AUDPC), severity, and vascular browning index of vascular wilt caused by *Fusarium oxysporum* f. sp. *physali* (Foph) in cape gooseberry seedlings with or without treatment using mixtures of BCAs and organic additives [Mix (*T. virens* + *B. velezensis*), MixRh (*T. virens* + *B. velezensis* + burnt rice husk) and MixChi (*T. virens* + *B. velezensis* + chitosan).

Treatment	AUDPC	Disease Severity Index	Vascular Browning
Foph^+^	69.3 a ^1^	3.20 a	4.80 a
Mix/Foph^+^	56.6 b	2.83 b	3.66 c
MixRh/Foph^+^	63.8 ab	2.97 ab	4.25 ab
MixChi/Foph^+^	57.6 b	2.95 ab	4.02 bc
Significance	** ^2^	*	***
CV (%) ^3^	7.28	4.67	6.47

^1^ Values followed by different letters in the same column are significantly different from *p* ≤ 0.05 according to the Tukey’s test; ^2^ *, ** and *** Significant at *p* ≤ 0.05, *p* ≤ 0.01 and *p* ≤ 0.01, respectively; ^3^ C.V.: Coefficient of variation.

## Data Availability

Not applicable.

## References

[1] Fischer G., Almanza-Merchán P.J., Miranda D. (2014). Importancia y cultivo de la uchuva (*Physalis peruviana* L.). Rev. Bras. Frutic..

[2] Puente L.A., Pinto-Muñoz C.A., Castro E.S., Cortés M. (2011). *Physalis peruviana* Linnaeus, the multiple properties of a highly functional fruit: A review. Food Res. Int..

[3] Bravo K., Osorio E. (2016). Characterization of polyphenol oxidase from Cape gooseberry (*Physalis peruviana* L.) fruit. Food Chem..

[4] Agronet. http://www.agronet.gov.co/estadistica/Paginas/default.aspx.

[5] Osorio-Guarín J.A., Enciso-Rodríguez F.E., González C., Fernández-Pozo N., Mueller L.A., Barrero L. (2016). Association analysis for disease resistance to *Fusarium oxysporum* in cape gooseberry (*Physalis peruviana* L). BMC Genom..

[6] Okungbowa F.I., Shittu H.O. (2012). *Fusarium* wilts: An overview. Environ. Res. J..

[7] Ochoa J., Clements C., Barrera V., Dominguez J.M., Ellis M.A., Alwang J., Muniappan R., Heinrichs E. (2016). IPM Packages for Naranjilla: Sustainable Production in an Environmentally Fragile Region. Integrated Pest Management of Tropical Vegetable Crops.

[8] Simbaqueba J., Catanzariti A., Gonzalez C., Jones D. (2018). Evidence for horizontal gene transfer and separation of effector recognition form effector function revealed by analysis of effector genes shared between cape-gooseberry and tomate-infecting formae speciales of *Fusarium oxysporum*. Mol. Plant Pathol..

[9] Enciso-Rodríguez F.E., González C., Rodríguez E.A., López C.E., Landsman D., Barrero L.S., Mariño-Ramírez L. (2013). Identification of immunity related genes to study the *Physalis peruviana*–*Fusarium oxysporum* pathosystem. PLoS ONE.

[10] Joshi R. (2018). A review of *Fusarium oxysporum* on its plant interaction and industrial use. J. Med. Plant Res..

[11] Moreno-Velandia C.A., Izquierdo-García L.F., Ongena M., Kloepper J.W., Cotes A.M. (2018). Soil sterilization, pathogen and antagonist concentration affect biological control of *Fusarium* wilt of cape gooseberry by *Bacillus velezensis* Bs006. Plant. Soil.

[12] Nogués S., Cotxarrera L., Alegre L., Trillas M.I. (2002). Limitations to photosynthesis in tomato leaves induced by *Fusarium* wilt. New Phytol..

[13] Dong X., Ling N., Wang M., Shen Q., Guo S. (2012). Fusaric acid is a crucial factor in the disturbance of leaf water imbalance in *Fusarium*-infected banana plants. Plant Physiol. Biochem..

[14] Wang M., Sun Y., Sun G., Liu X., Zhai L., Shen Q., Guo S. (2015). Water balance altered in cucumber plants infected with *Fusarium oxysporum* f. sp. *cucumerinum*. Sci. Rep..

[15] Sun Y., Wang M., Li Y., Gu Z., Ling N., Shen Q., Guo S. (2017). Wilted cucumber plants infected by *Fusarium oxysporum* f. sp. *Cucumerinum* do not suffer from water shortage. Ann. Bot..

[16] Morkunas I., Bednarski W. (2008). *Fusarium oxysporum* induced oxidative stress and antioxidative defenses of yellow lupine embryo axes with different level of sugars. J. Plant Physiol..

[17] De Menezes H.D., Tonani L., Bachmann L., Wainwright M., Braga G.Ú.L., von Zeska Kress M.R. (2016). Photodynamic treatment with phenothiazinium photosensitizers kills both ungerminated and germinated microconidia of the pathogenic fungi *Fusarium oxysporum*, *Fusarium moniliforme* and *Fusarium solani*. J. Photochem. Photobiol. B Biol..

[18] Gordon T.R. (2017). *Fusarium oxysporum* and the *Fusarium* wilt syndrome. Annu. Rev. Phytopathol..

[19] Chávez-Arias C.C., Gómez-Caro S., Restrepo-Díaz H. (2019). Physiological, Biochemical and Chlorophyll Fluorescence Parameters of *Physalis Peruviana* L. Seedlings Exposed to Different Short-Term Waterlogging Periods and *Fusarium* Wilt Infection. Agronomy.

[20] McGovern R.J. (2015). Management of tomato diseases caused by *Fusarium oxysporum*. Crop. Prot..

[21] Pal K.K., Mc Spadden Gardener B. (2006). Biological Control of Plant Pathogens. Plant Health Instr..

[22] Köhl J., Kolnaar R., Ravensberg W.J. (2019). Mode of action of microbial biological control agents against plant diseases: Relevance beyond efficacy. Front. Plant Sci..

[23] Ab Rahman S.F.S., Singh E., Pieterse C.M., Schenk P.M. (2018). Emerging microbial biocontrol strategies for plant pathogens. Plant Sci..

[24] O’Brien P.A. (2017). Biological control of plant diseases. Australas. Plant Pathol..

[25] Xu X.M., Jeffries P., Pautasso M., Jeger M.J. (2011). Combined use of biological control agents to management plant diseases in theory and practice. Phytopathology.

[26] Izquierdo-García L.F., González-Almario A., Cotes A.M., Moreno-Velandia C.A. (2020). *Trichoderma virens* Gl006 and *Bacillus velezensis* Bs006: A compatible interaction controlling *Fusarium* wilt of cape gooseberry. Sci. Rep..

[27] Xu X., Robinson J., Jeger M., Jeffries P. (2010). Using combinations of biocontrol agents to control *Botrytis cinerea* on strawberry leaves under fluctuating temperatures. Biocontrol Sci. Technol..

[28] Howell C.R. (2003). Mechanisms employed by *Trichoderma* species in the biological control of plant diseases: The history and evolution of current concepts. Plant Dis..

[29] Cawoy H., Bettiol W., Fickers P., Onge M. (2011). Bacillus-Based Biological Control of Plant Diseases. Pesticides in the Modern World Pesticides Use and Management.

[30] López-Bucio J., Pelagio-Flores R., Herrera-Estrella A. (2015). *Trichoderma* as biostimulant: Exploiting the multilevel properties of a plant beneficial fungus. Sci. Hortic..

[31] Waghunde R.R., Shelake R.M., Sabalpara A.N. (2016). *Trichoderma*: A significant fungus for agriculture and environment. Afr. J. Agric. Res..

[32] Shafi O., Tian H., Ji M. (2017). *Bacillus* species as versatile weapons for plant pathogens: A review. Biotechnol. Biotechnol. Equip..

[33] Pedraza L.A., López C.E., Uribe-Velez D. (2020). Mechanisms of action of *Bacillus* spp. (Bacillaceae) against phytopathogenic microorganisms during their interaction with plants. Acta Biol. Colomb..

[34] Howell C.R. (2006). Understanding the mechanisms employed by *Trichoderma virens* to effect biological control of cotton diseases. Phytopathology.

[35] Jaimes Suárez Y.Y., Moreno Velandia C.A., Cotes Prado A.M. (2009). Induced Systemic Resistance Against *Fusarium oxysporum* In Tomato by *Trichoderma koningiopsis* Th003. Acta Biol. Colomb..

[36] Wang B.B., Yuan J., Zhang J., Shen Z.Z., Zhang M.X., Li R., Ruan Y.Z., Shen Q.R. (2013). Effects of novel bioorganic fertilizer produced by *Bacillus amyloliquefaciens* W19 on antagonism of *Fusarium* wilt of banana. Biol. Fertil. Soils.

[37] Yuan J., Ruan Y., Wang B., Zhang J., Waseem R., Huang Q., Shen Q. (2013). Plant growth-promoting rhizobacteria strain *Bacillus amyloliquefaciens* NJN-6-enriched bio-organic fertilizer suppressed *Fusarium* wilt and promoted the growth of banana plants. J. Agric. Food Chem..

[38] Chaves-Gómez J.L., Chavez-Arias C.C., Cotes Prado A.M., Gómez-Caro S., Restrepo-Díaz H. (2019). Physiological response of cape gooseberry seedlings to three biological control agents under *Fusarium oxysporum* f. sp. *physali* Infection. Plant Dis..

[39] El Hadrami A., Adam L.R., El Hadrami I., Daayf F. (2010). Chitosan in Plant Protection. Mar. Drugs.

[40] Hassan O., Chang T. (2017). Chitosan for eco-friendly control of plant disease. Asian J. Plant Pathol..

[41] Ways T.M.M., Lau W.M., Khutoryanskiy V.V. (2018). Chitosan and Its Derivatives for Application in Mucoadhesive Drug Delivery Systems. Polymers.

[42] Chakraborty M., Hasanuzzaman M., Rahman M., Khan M.A.R., Bhowmik P., Mahmud N.U., Tanveer M., Islam T. (2020). Mechanism of Plant Growth Promotion and Disease Suppression by Chitosan Biopolymer. Agriculture.

[43] Al-Hetar M.Y., Zainal Abidin M.A., Sariah M., Wong M.Y. (2011). Antifungal activity of chitosan against *Fusarium oxysporum* f. sp. *cubense*. J. Appl. Polym. Sci..

[44] Berger L.R.R., Stamford N.P., Willadino L.G., Laranjeira D., de Lima M.A.B., Malheiros S.M.M., de Oliveira W.J., Stamford T.C.M. (2016). Cowpea resistance induced against *Fusarium oxysporum* f. sp. tracheiphilum by crustaceous chitosan and by biomass and chitosan obtained from Cunninghamella elegans. Biol. Control.

[45] Chaves-Gómez J.L., Cotes-Prado A.M., Gómez-Caro S., Restrepo-Díaz H. (2020). Physiological response of cape gooseberry seedlings to two organic additives and their mixture under inoculation with *Fusarium oxysporum* f. sp. *physali*. HortScience.

[46] Bonanomi G., Ippolito F., Scala F. (2015). A black future for plant pathology? Biochar as a new soil amendment for controlling plant diseases. J. Plant Pathol..

[47] Eo J., Park K.-C., Kim M.-H., Kwon S.-I., Song Y.-J. (2018). Effects of rice husk and rice husk biochar on root rot disease of ginseng (*Panax ginseng*) and on soil organisms. Biol. Agric. Hortic..

[48] Somapala K., Weerahewa D., Thrikawala S. (2016). Silicon rich rice hull amended soil enhances anthracnose resistance in tomato. Proced. Food Sci..

[49] Ratnayake R.M.R.N.K., Ganehenege M., Ariyarathne H., Daundasekera W. (2018). Soil application of rice husk as a natural silicon source to enhance some chemical defense responses against foliar fungal pathogens and growth performance of Bitter Gourd (*Momordica charantia* L.). Ceylon J. Sci..

[50] Sid Ahmed A., Ezziyyani M., Pérez Sánchez C., Candela M.E. (2003). Effect of chitin on biological control activity of *Bacillus* spp. and *Trichoderma harzianum* against root rot disease in pepper (*Capsicum annuum*) plants. Eur. J. Plant Pathol..

[51] Nitu N.J., Masum M.I., Jannat R., Sultana S., Bhuiyan K.A. (2016). Application of chitosan and *Trichoderma* against soil-borne pathogens and their effect on yield of tomato (*Solanum lycopersicum* L.). Int. J. Biosci..

[52] Benhamou N., Kloepper J.W., Tuzun S. (1998). Induction of resistance against *Fusarium* wilt of tomato by combination of chitosan with an endophytic bacterial strain: Ultrastructure and cytochemistry of the host response. Planta.

[53] Hashem A., Tabassum B., Abd_Allah E.F. (2019). *Bacillus subtilis*: A plant-growth promoting rhizobacterium that also impacts biotic stress. Saudi J. Biol. Sci..

[54] Mendoza-Vargas L.A., Villamarín-Romero W.P., Cotrino-Tierradentro A.S., Ramírez-Gil J.G., Chávez-Arias C.C., Restrepo-Díaz H., Gómez-Caro S. (2021). Physiological Response of Cape Gooseberry Plants to *Fusarium oxysporum* f. sp. *physali*, Fusaric Acid, and Water Deficit in a Hydrophonic System. Front. Plant Sci..

[55] Christopher D.J., Raj T.S., Rani S.U., Udhayakumar R. (2010). Role of defense enzymes activity in tomato as induced by *Trichoderma virens* against *Fusarium* wilt caused by *Fusarium oxysporum* f.sp. *lycopersici*. J. Biopestic..

[56] Bell A.A., Hubbard J.C., Liu L., Davis R.M., Subbarao K.V. (1998). Effects of chitin and chitosan on the incidence and severity of *Fusarium* yellows in celery. Plant Dis..

[57] Casals C., Elmer P.A.G., Viñas I., Teixidó N., Sisquella M., Usall J. (2012). The combination of curing with either chitosan or *Bacillus subtilis* CPA-8 to control brown rot infections caused by *Monilinia fructicola*. Postharvest Biol. Technol..

[58] El-Mohamedy R.S.R., Abdel-Kareem F., Jabnoun-Khiareddine H., Daami-Remadi M. (2014). Chitosan and *Trichoderma harzianum* as fungicide alternatives for controlling *Fusarium* crown and root rot of tomato. Tunis. J. Plant Prot..

[59] Bakeer A.R.T., El-Mohamedy R.S., Saied N.M., Abd-El-Kareem F. (2016). Field Suppression of *Fusarium* Soil Borne Diseases of Tomato Plants by the Combined Application of Bio Agents and Chitosan. Br. Biotechnol. J..

[60] Ruano-Rosa D., Mercado-Blanco J., Meghvansi M.K., Varma A. (2015). Combining biocontrol agents and organics amendments to manage soil-borne phytopathogens. Organic Amendments and Soil Suppressiveness in Plant Disease Management.

[61] Okorski A., Olszewski J., Głowacka K., Okorska S., Pszczółkowska A. (2012). The effect of the application of the biological control agent EM1 on gas exchange parameters and productivity of *Pisum sativum* L. infected with *Fusarium oxysporum* Schlecht. ACTA Agrobot..

[62] Pantelides I., Tjamos S., Pappa S., Kargakis M., Paplomatas E.J. (2013). The ethylene receptor ETR1 is required for *Fusarium oxysporum* pathogenicity. Plant Pathol..

[63] Zaim S., Bekkar A.A., Belabid L. (2018). Efficacy of *Bacillus subtilis* and *Trichoderma harzianum* combination on chickpea *Fusarium* wilt caused by *F. oxysporum* f. sp. *ciceris*. J. Plant Prot. Res..

[64] Ayala A., Muñoz M.F., Argüelles S. (2014). Lipid peroxidation: Production, metabolism, and signaling mechanisms of malondialdehyde and 4-hydroxy-2-nonenal. Oxid. Med. Cell Longev..

[65] Dar M.I., Naikoo M.I., Rehman F., Naushin F., Khan F.A. (2016). Proline accumulation in plants: Roles in stress tolerance and plant development. Osmolytes and Plants Acclimation to Changing Environment: Emerging Omics Technologies.

[66] Pérez-Bueno M.L., Pineda M., Barón M. (2019). Phenotyping plant responses to biotic stress by chlorophyll fluorescence imaging. Front. Plant Sci..

[67] Alamri S.A. (2014). The synergistic effect of two formulated biofungicides in the biocontrol of root and bottom rot of lettuce. Biocontrol Sci..

[68] Mishra S., Jagadeesh K.S., Krishnaraj P.U., Prem S. (2014). Biocontrol of tomato leaf curl virus (ToLCV) in tomato with chitosan supplemented formulations of *Pseudomonas* sp. under field conditions. Aust. J. Crop Sci..

[69] Zhang H.Y., Ge L.L., Chen K.P., Zhao L.N., Zhang X.Y. (2014). Enhanced biocontrol activity of *Rhodotorula mucilaginosa* cultured in media containing chitosan against postharvest diseases in strawberries: Possible mechanisms underlying the effect. J. Agric. Food Chem..

[70] Mukherjee M., Mukherjee P.K., Horwitz B.A., Zachow C., Berg G., Zeilinger S. (2012). Trichoderma–Plant–Pathogen Interactions: Advances in Genetics of Biological Control. Indian J. Microbiol..

[71] Nusaibah S.A., Musa H., Mohammad M.S., Sharif U., Buhari T.R. (2019). A Review report on the mechanism of *Trichoderma* spp. as biological control agent of the Basal Stem Rot (BSR) disease of *Elaeis guineensis*. Trichoderma—The Most Widely Used Fungicide.

[72] Fan B., Wang C., Song X.F., Ding X.L., Wu L.M., Wu H.J., Gao X.W., Borriss R. (2018). *Bacillus velezensis* FZB42 in 2018: The gram-positive model strain for plant growth promotion and biocontrol. Front. Microbiol..

[73] Gowtham H.G., Murali M., Singh S.B., Lakshmeesha T.R., Murthy K.N., Amruthesh K.N., Niranjana S.R. (2018). Plant growth promoting rhizobacteria *Bacillus amyloliquefaciens* improves plant growth and induces resistance in chilli against anthracnose disease. Biol. Control.

[74] Rabbee M.F., Ali M.S., Choi J., Hwang B.S., Jeong S.C., Baek K.-h. (2019). *Bacillus velezensis:* A Valuable Member of Bioactive Molecules within Plant Microbiomes. Molecules.

[75] Gül A., Kidoglu F., Tüzel Y., Tüzel I.H. (2008). Effects of nutrition and “Bacillus amyloliquefaciens” on tomato (“*Solanum lycopersicum* L.”) growing in perlite. Span. J. Agric. Res..

[76] Vieira P.M., Santos M.P., Andrade C.M., Souza-Neto O.A., Ulhoa C.J., Aragao F.J.L. (2017). Overexpression of an aquaglyceroporin gene from *Trichoderma harzianum* improves water-use efficiency and drought tolerance in *Nicotiana tabacum*. Plant Physiol. Biochem..

[77] Kumar S.M., Chowdappa P., Krishna V. (2015). Development of seed coating formulation using consortium of *Bacillus subtilis* OTPB1 and *Trichoderma harzianum* OTPB3 for plant growth promotion and induction of systemic resistance in field and horticultural crops. Indian Phytopath..

[78] Zeilinger S., Gruber S., Bansal R., Mukherjee P.K. (2016). Secondary metabolism in *Trichoderma*—Chemistry meets genomics. Fungal Biol. Rev..

[79] Doni F., Zain C.R.C.M., Isahak A., Fathurrahman F., Sulaiman N., Uphoff N., Yusoff W.M.W. (2017). Relationships observed between Trichoderma inoculation and characteristics of rice grown under System of Rice Intensification (SRI) vs. conventional methods of cultivation. Symbiosis.

[80] Zehra A., Meena M., Dubey M.K., Aamir M., Upadhyay R.S. (2017). Synergistic effects of plant defense elicitors and *Trichoderma harzianum* on enhanced induction of antioxidant defense system in tomato against *Fusarium* wilt disease. Bot. Stud..

[81] Karuppiah V., Li T., Vallikkannu M., Chen J. (2019). Co-cultivation of *Trichoderma asperellum* GDFS1009 and *Bacillus amyloliquefaciens* 1841 causes differential gene expression and improvement in the wheat growth and biocontrol activity. Front. Microbiol..

[82] Kheiri A., Moosawi Jorfa S.A., Malihipourb A., Saremic H., Nikkhahda M. (2017). Synthesis and characterization of chitosan nanoparticles and their effect on *Fusarium* head blight and oxidative activity in wheat. Int. J. Biol. Macromol..

[83] Bistgani Z.E., Siadat S.A., Bakhshandeh A., Pirbalouti A.G., Hashemic M. (2017). Interactive effects of drought stress and chitosan application on physiological characteristics and essential oil yield of *Thymus daenensis* Celak. Crop J..

[84] Rahman M., Mukta J.A., Sabir A.A., Gupta D.R., Mohi-ud-din M., Hasanuzzaman M., Miah M.G., Rahman M., Islam M.T. (2018). Chitosan biopolymer promotes yield and stimulates accumulation of antioxidants in strawberry fruit. PLoS ONE.

[85] Xu C., Mou B. (2018). Chitosan as Soil Amendment Affects Lettuce Growth, Photochemical Efficiency, and Gas Exchange. HortTechnology.

[86] Muley A.B., Shingote P.R., Patil A.P., Dalvi S.G., Suprasanna P. (2019). Gamma radiation degradation of chitosan for application in growth promotion and induction of stress tolerance in potato (*Solanum tuberosum* L.). Carbohydr. Polym..

[87] Rabêlo V.M., Magalhães P.C., Bressanin L.A., Carvalho D.T. (2019). The foliar application of a mixture of semisynthetic chitosan derivatives induces tolerance to water deficit in maize, improving the antioxidant system and increasing photosynthesis and grain yield. Sci. Rep..

[88] Araujo A.S.D., Blum L.E.B., Figueiredo C.C.D. (2019). Biochar and *Trichoderma harzianum* for the Control of *Macrophomina phaseolina*. Braz. Arch. Biol. Technol..

[89] Akhter A., Hage-Ahmed K., Soja G., Steinkellner S. (2016). Potential of *Fusarium* wilt-inducing chlamydospores, in vitro behaviour in root exudates and physiology of tomato in biochar and compost amended soil. Plant Soil.

[90] Win K.T., Okazaki K., Ookawa T., Yokoyama T., Ohwaki Y. (2019). Influence of rice-husk biochar and Bacillus pumilus strain TUAT-1 on yield, biomass production, and nutrient uptake in two forage rice genotypes. PLoS ONE.

[91] Olivares B.O., Araya-Alman M., Acevedo-Opazo C., Rey J.C., Cañete-Salinas P., Kurina F.G., Balzarini M., Lobo D., Navas-Cortés J.A., Landa B.B. (2020). Relationship between soil properties and banana productivity in the two main cultivation areas in venezuela. J. Soil Sci. Plant.

[92] Olivares B.O., Rey J.C., Lobo D., Navas-Cortés J.A., Gómez J.A., Landa B.B. (2021). *Fusarium* Wilt of Bananas: A Review of Agro-Environmental Factors in the Venezuelan Production System Affecting Its Development. Agronomy.

[93] Olivares Campo B., Paredes F., Rey J., Lobo D., Galvis-Causil S. (2021). The relationship between the normalized difference vegetation index, rainfall, and potential evapotranspiration in banana plantation of Venezuela. Sains Tanah J. Soil Sci. Agroclimatol..

[94] Park D. (1961). Isolation of *Fusarium oxysporum* from soils. Trans. Br. Mycol. Soc..

[95] Moreno-Velandia C. (2018). Interactions between *Bacillus amyloliquefaciens* Bs006, *Fusarium oxysporun* Map5 and Cape Gooseberry (*Physalis peruviana*). Ph.D. Thesis.

[96] Chiang K.S., Liu H.I., Tsai J.W., Tsai J.R., Bock C.H. (2017). A discussion on disease severity index values. Part II: Using the disease severity index for null hypothesis testing. Ann. Appl. Biol..

[97] Campbell C.L., Madden L.V. (1990). Introduction to Plant Disease Epidemiology.

[98] Leslie J.F., Summerell B.A. (2006). The Fusarium Laboratory Manual.

[99] Mandal S., Mallick N., Mitra A. (2009). Salicylic acid-induced resistance to *Fusarium oxysporum* f. sp. *lycopersici* in tomato. Plant Physiol. Biochem..

[100] Abbott W. (1925). A method of computing the effectiveness of an insecticide. J. Econ. Entomol..

[101] Roussos A.P., Denaxa N.K., Damvakaris T., Stournaras V., Argyrokastritis I. (2010). Effect of alleviating products with different mode of action on physiology and yield of olive under drought. Sci. Hort..

[102] Lichtenthaler H.K. (1987). Chlorophylls and carotenoids: Pigments of photosynthetic biomembrans. Methods Enzymol..

[103] Hodges D.M., DeLong J.M., Forney C.F., Prange R.K. (1999). Improving the thiobarbituric acid-reactive-substances assay for estimating lipid peroxidation in plant tissues containing anthocyanin and other interfering compounds. Planta.

[104] Bates L.S., Waldren R.P., Teare I.D. (1973). Rapid determination of free proline for water-stress studies. Plant. Soil.

